# Homeostatic capabilities of the choroid plexus epithelium in Alzheimer's disease

**DOI:** 10.1186/1743-8454-1-3

**Published:** 2004-12-10

**Authors:** Conrad Johanson, Paul McMillan, Rosemarie Tavares, Anthony Spangenberger, John Duncan, Gerald Silverberg, Edward Stopa

**Affiliations:** 1 Department of Clinical Neurosciences, Brown Medical School, Providence, RI 02903, USA; 2 Department of Pathology, Brown Medical School, Providence, RI 02903,USA

## Abstract

As the secretory source of vitamins, peptides and hormones for neurons, the choroid plexus (CP) epithelium critically provides substances for brain homeostasis. This distributive process of cerebrospinal fluid (CSF) volume transmission reaches many cellular targets in the CNS. In ageing and ageing-related dementias, the CP-CSF system is less able to regulate brain interstitial fluid. CP primarily generates CSF bulk flow, and so its malfunctioning exacerbates Alzheimers disease (AD). Considerable attention has been devoted to the blood-brain barrier in AD, but more insight is needed on regulatory systems at the human blood-CSF barrier in order to improve epithelial function in severe disease. Using autopsied CP specimens from AD patients, we immunocytochemically examined expression of heat shock proteins (HSP90 and GRP94), fibroblast growth factor receptors (FGFr) and a fluid-regulatory protein (NaK2Cl cotransporter isoform 1 or NKCC1). CP upregulated HSP90, FGFr and NKCC1, even in end-stage AD. These CP adjustments involve growth factors and neuropeptides that help to buffer perturbations in CNS water balance and metabolism. They shed light on CP-CSF system responses to ventriculomegaly and the altered intracranial pressure that occurs in AD and normal pressure hydrocephalus. The ability of injured CP to express key regulatory proteins even at Braak stage V/VI, points to plasticity and function that may be boosted by drug treatment to expedite CSF dynamics. The enhanced expression of human CP 'homeostatic proteins' in AD dementia is discussed in relation to brain deficits and pharmacology.

## Review

### Choroid plexus impact on Alzheimer's disease

Accumulating evidence supports the idea that continually decreasing choroid plexus (CP) function in advanced ageing exacerbates Alzheimer's disease (AD). As part of a new paradigm to explain brain interstitium deterioration in age-related dementias, increasingly more attention is being paid to the role of compromised blood-CSF [1] and blood-brain [2] barriers. Structural alterations and functional failures in CP as well as brain capillary transport systems adversely affect fluid dynamics and composition [3,4]. This review treats mainly CP dysfunction and the compensatory reactions that occur in this epithelium in AD.

Efficient CSF turnover is essential for a healthy brain. It depends upon an exquisite balance between CSF formation and reabsorption [5]. Compromised secretory phenomena at the CP 'upstream' predispose the brain to AD-type problems. On the other hand, defective clearance of CSF at the arachnoid membrane 'downstream' leads to normal pressure hydrocephalus (NPH) [6]. The pivotal role of the CP in CSF homeostasis and brain viability becomes more evident when the system fails. More information is needed to evaluate how diminishing choroidal functions affect the surrounding brain in the face of AD and other dementias.

### Brain fluid homeostasis: The role of CSF volume transmission

**Figure 1:**
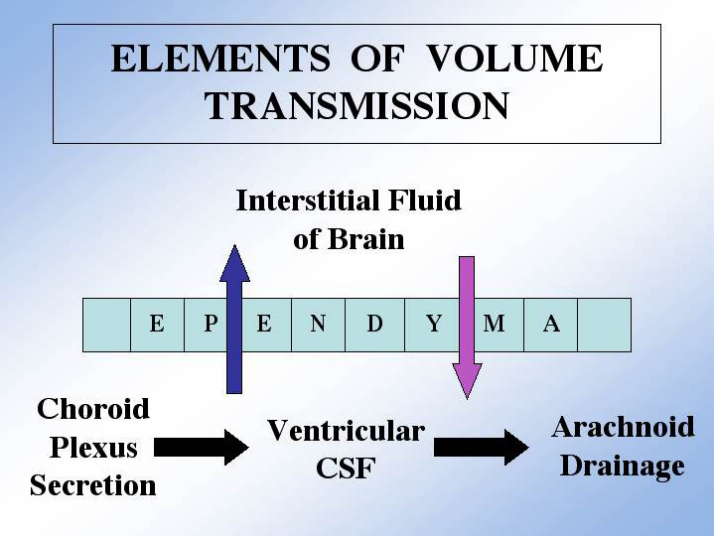
Schema for CSF convection of water and solutes: Arterial blood perfusing the choroid plexus continually provides water, ions and organic substrates for the CP epithelial cells to form the CSF that underlies volume transmission. Manufactured by the CP epithelium as the result of numerous transport processes, the CSF is actively secreted into the ventricles. In transit through the ventricular cavities to the arachnoid drainage sites, the CSF exchanges anabolites and catabolites with brain interstitial fluid. As a result, trophic and signaling molecules are delivered (blue arrow) to the neurons and, concurrently, toxic waste products and unneeded proteins are removed (red arrow) by CSF 'sink action' on the brain. The permeable ependymal membrane allows bi-directional diffusion of beneficial and harmful molecules. Bulk flow or volume transmission of CSF is thus essential in effecting the homeostasis of fluid composition.

In serving the brain's metabolic needs by supplying 'biochemical goods', the CP uses CSF as a conduit for convecting substances [7]. Numerous solutes ranging from ions to large proteins are entrained in the CSF that percolates through the ventricular axis (Fig. 1). CSF intimately contacts the periventricular brain tissue with which it exchanges materials bidirectionally by diffusion and bulk flow. Continually undergoing chemical modification as it flows downstream, the CSF completes the volume transmission process [7] by draining into venous blood at distal arachnoidal sites (Fig. 1).

Reduced formation of CSF and stagnated flow in ageing and AD [4] limits the delivery of substances to neurons. This debilitates brain function [3]. Numerous proteins are synthesized and secreted by CP into CSF. Other substances are transported from blood. Table 1 overviews molecules normally distributed by the CP-CSF nexus. Arginine vasopressin (AVP) is a neuropeptide synthesized by CP epithelium and secreted into CSF [8,9]; it regulates CSF formation and modulates hippocampal memory mechanisms [10,11]. Growth factors foster cell growth, blood supply and water balance [1]. Trace element distribution across CP is complex and involves many mechanisms. Iron transport into CSF, for example, is regulated by several proteins [1] alterations in which may predispose to amyloidogenesis [12]. Vitamins B and C are actively transported at the blood-CSF barrier [13]. This stabilizes their concentration in CSF, except in late-onset AD [14]. The net entry of nucleoside bases into CSF is also determined by active transporters in CP [15,16]. Cysteine protease inhibitors like cystatin C are synthesized in CP epithelium for transport into CSF [17]. Hormones such as leptin and prolactin are translocated from plasma to CSF by saturable choroidal receptors [18,19]. Overall it is striking that the CP-CSF interface engages in such prolific transport. Disease-associated interference with CP transporters and volume transmission limits the availability of CSF molecules for the brain [1,20].

**Table 1:** Substances distributed to brain by transport at the blood-CSF gateway

Class or group	Examples	Functions	References
Neuropeptides	Arginine vasopressin	Regulation of CSF formation	[8–11]
Growth factors	Basic fibroblast growth factor	Integration of water balance	[55, 104]
Trace elements	Iron	Enzyme function in neurons/glia	[1, 12]
Vitamins	Ascorbate & folate	Antioxidants & co-factors for brain	[13, 14]
Nucleoside bases	Thymidine	Nucleic acids & drug transport	[15, 16]
Protease inhibitors	Cystatin C	Neuroprotection after ischemia	[1, 17]
Hormones	Prolactin & leptin	Modulation of hypothalamus	[18, 19]

### Structural and functional damage to the choroid plexus in AD

Structure intimately relates to function in various epithelia. Therefore it is useful to analyze histopathological damage to CP at various stages of AD in order to clarify the onset of functional losses at the blood-CSF barrier. Ageing and AD cause similar degeneration in CP. Structural changes in AD though are usually greater than in non-demented control counterparts [21]. Serot and colleagues have delineated modifications in the choroidal epithelium, stroma and vessels in AD subjects [22-24]. There are substantial alterations in all tissue compartments.

Several features characterizing the CP in AD are highlighted in Table 2. Epithelial cells are typically truncated, with an average volume 70% of that at birth. Such epithelial atrophy likely affects cellular functions. Particularly prominent is an increase in the number of Biondi bodies in AD [25]. These bodies are fibrillar inclusions in the cytoplasm of very old epithelial cells. Lipofuchsin vacuoles also occur frequently in the cytoplasm. The basement membrane underlying the cell often thickens to 350 nm, compared to a much thinner membrane (ca. 100 nm) in neonates [21]. Another liability in AD is immunological deposition of C1q, IgG and IgM along the epithelial basement membrane. As fibrosis intensifies with age and disease, the stroma attains a thickness of a few tenths of a micron [21]. At the inner core of the choroidal villus, the blood vessel walls thicken. This coincides with the appearance of amyloid, hyaline bodies, psammomas and calcifications. Altogether, the histopathologic changes in CP compartments point to grossly-declining secretory functions in AD. Such structural abnormalities coincide with diminished CSF production in ageing [26] and AD patients [4].

**Table 2:** Pathological changes in choroid plexus in Alzheimer's disease^a^

Epithelial atrophy (↓ cell size by 1/3)
↑ Biondi bodies
Lipofuchsin vacuoles
Basement membrane thickening (3-fold ↑)
↑ Stromal fibrosis
IgG and IgM depositions

^a^Described by Serot and colleagues in refs. 22–24

Due to the functional nexus of the CSF with brain interstitial fluid, the neuronal microenvironment in AD is impacted by markedly altered transport and permeability in CP. When neurodegenerative diseases, ischemia [27,28] and elevated pressure [29,30] inflict damage on the choroidal epithelium, there are resultant adverse changes in CSF composition and volume. Because neurons are sensitive to instabilities in CSF dynamics and constituents, it is important to assess the nature and progression of disrupted CP function in chronic diseases. In view of the expanding number of AD victims, it is timely to consider patterns of expression of chaperone proteins, receptors and transporters in CP at various Braak stages. Such information may abet future attempts to stabilize choroid plexus functional integrity perturbed in early AD dementia.

### Choroid plexus defense against insults in ageing and degeneration

The CP is multifunctional, performing a wide range of homeostatic functions for the CNS [5]. CSF homeostasis is mediated mainly by CP. It involves the activity of many protein transporters and receptors at the basolateral and apical surfaces of the epithelial cells [31]. In addition to providing organic solutes for nutritive and trophic support of the brain, the CP secretions into the ventricles adjust the pH, osmolality, [K^+^] and immune molecule content of the CNS extracellular fluid [7]. Accordingly, healthy neurons are fundamentally dependent upon transport at the blood-CSF and blood-brain interfaces.

Moreover to provide steady neuroprotection by regulating the extracellular milieu, the CP must protect itself against various stressor agents that build up during ageing and disease. There have been few investigations of the homeostatic systems within CP that stabilize choroidal functions in the face of ageing and AD. We have explored some candidate systems for compensatory responses by CP. Cytoplasmic heat shock proteins chaperone and perform housekeeping to maintain a healthy steady-state intraepithelial milieu [1,32]. Growth factors critically minimize cell morbidity and mortality [27]. Fluid-regulating proteins correct ion and water imbalances that occur in neurodegenerative diseases. Consequently our hypothesis is that the upregulation of certain proteins in CP (such as those discussed above) thwarts certain untoward effects of ageing and disease progression. The protein expression aspect of the hypothesis was tested by analyzing immunostaining patterns in human CP specimens from patients with varying severity of AD.

### Analyses of human choroid plexus: Usefulness and challenges

It is difficult to functionally assess the CP in vivo [30,33], particularly in man. Alternatively one can evaluate the status of human CP in disease by analyzing autopsied tissues for variable protein expression [34]. Such findings are compared to appropriate age-matched controls. Immumocytochemical and biochemical data gleaned from human CP highlight directions to pursue with living animal models: transgenic mice with an AD phenotype or aged rats with CSF pathophysiology. Because protein expression relates to disease progression, it is essential to standardize grading for the severity of AD in subjects. We use a modified Braak & Braak staging system [35]: stages I/II (mild; disease involves hippocampus and entorhinal cortex); stages III/IV (moderate; AD spreading to the rest of the limbic lobe, e.g., amygdala); and stages V/VI (severe; further AD spreading to the prefrontal neocortex).

Information about functional proteins in human CP is scarce. However protein expression was recently analyzed in autopsied lateral ventricle CP from normal adult brains and in those with confirmed AD [36,37]. The ages investigated were generally between 65 and 90 yr for all subjects. Control individuals typically died from cardiac disease or tumors. AD specimens covered all Braak stages. Causes of the AD deaths were usually cardiac complications or pneumonia [37]. Fortunately the postmortem intervals (mainly between 2 and 18 hr) did not affect antibody staining [37]. For specimen quality it is desirable to procure choroidal tissues quickly after death. Human CP banks are needed to systematically catalog tissues from various stages of AD.

### Regulation of CP 'homeostatic proteins' in health and disease

Ageing and AD dementia tax the CP and other CNS transport interfaces. In late life the deteriorating brain presents many potentially-destructive metabolites to the CSF for multi-site excretion into blood. The greater burden of macromolecule disposal in AD occurs when CP and arachnoid membrane, due to ageing debilities, are less able to transfer solutes. Nevertheless the CP seemingly attempts to maintain its 'epithelial soundness' when challenged to perform additional cleansing acts for the brain extracellular fluid (CSF).

In healthly, young adults the CP epithelium sensitively acclimates to chemical and physical distortions in blood, CSF and parenchyma. Following cortical stabbing in rats, the CP upregulates TGFβ presumably to provide this CSF-borne growth factor for repairing the injury [38]. A similar phenomenon occurs with IGF-II [39], which is manufactured in the CNS mainly by CP. In diabetes there is enhanced expression of the NKCC1 cotransporter in CP for adjusting CSF dynamics and water distribution [40]. Such compensatory responses at the blood-CSF barrier in early adulthood raise the question about CP's ability in later life, when besieged by the deficits of aging and AD, to adequately respond by expressing certain 'homeostatic proteins'.

Accordingly to assess pathophysiologic consequences of AD we investigated human CP's ability to upregulate certain functional proteins (as distinguished from structural ones) in advanced states of AD dementia. With advancing knowledge about intricate cell physiology (coordinated interactions among organelles, cytoplasm and membrane-bound proteins) it is relevant to evaluate components of cellular homeostasis. The term 'homeostatic proteins'refers to chaperones, receptors and transporters that stabilize the internal environment of the cell. CSF stability depends in large part upon CP epithelial homeostasis. Our group has analyzed several 'homeostatic proteins' involved in CP intracellular milieu stabilization:

#### Heat shock proteins

A wide spectrum of protection against neurodegeneration is provided by HSPs. The list of protective effects bestowed by HSP molecules is diverse and includes: accelerated degradation of misfolded proteins, maintenance of membrane lipid integrity, prevention of deleterious protein aggregation, and preclusion of damage to the translational apparatus [41,42]. HSPs are also known as 'stress proteins' due to their role in shepherding adaptive responses to stressors such as ischemia, trauma, fever, dehydration, hydrocephalus and other brain disorders.

**Figure 2:**
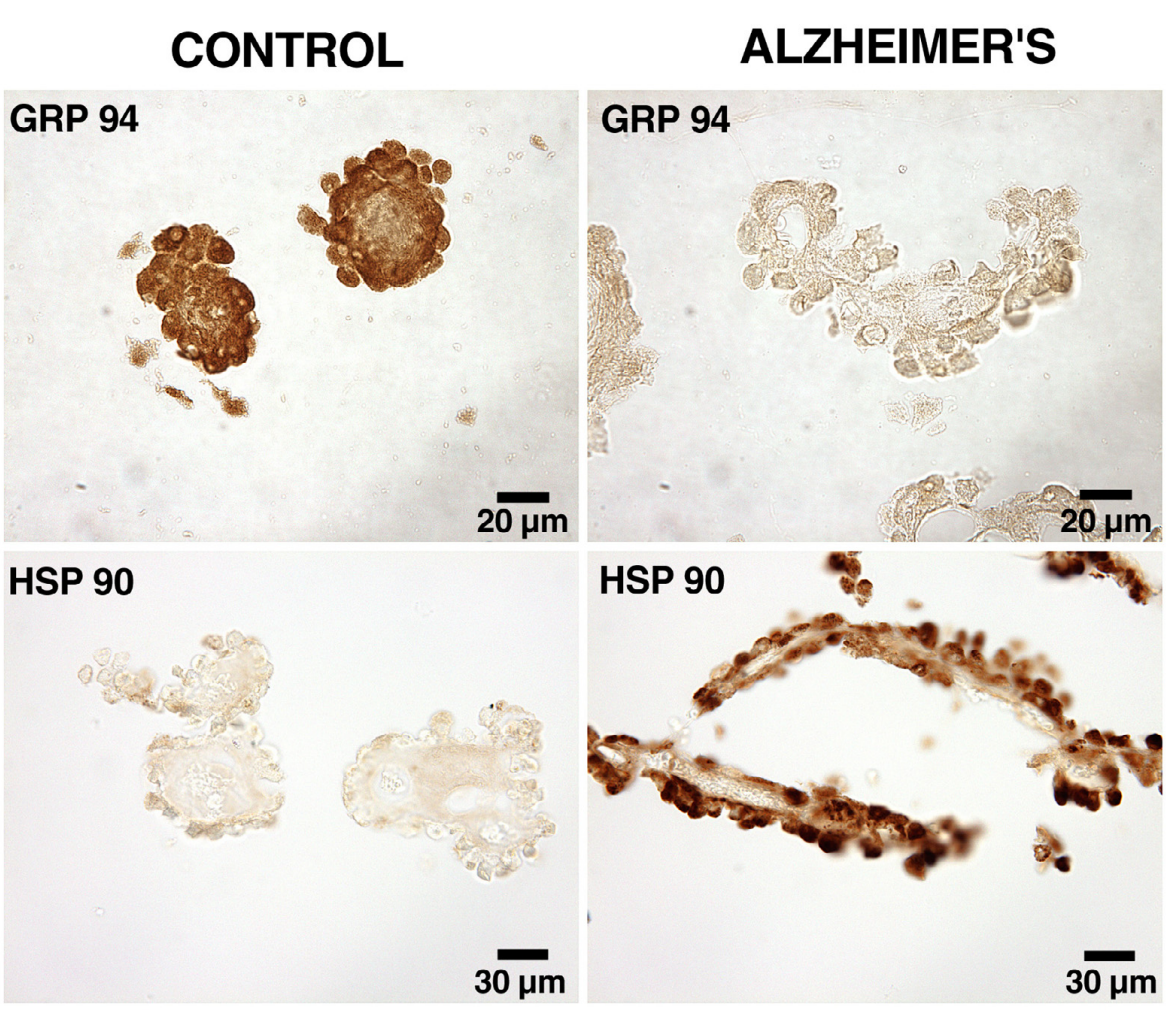
Heat shock (stress) protein expression in human CP: Formalin-fixed, paraffin-embedded specimens (8–10 micrometers thick) were de-paraffinized and rehydrated. Sections were incubated overnight with antibodies against HSP90 (1/500; SPA830) and GRP94 (1/500;SPA850) and stained by the ABC technique (Vectastain Elite ABC peroxidase). Deposition of the brown chromogen (diaminobenzidine) reaction product was either substantially reduced by preabsorption blocking or virtually eliminated by omission of either primary or secondary antibodies. Slides were assessed blindly for staining intensity and distribution [37]. See text for description of localization and interpretation. For each HSP, images are representative of 10 AD specimens and 5 age-matched controls.

To evaluate human CP expression, we selected HSPs overexpressed in AD brains: GRP94 and HSP90. In contrast to previously found upregulation in brain, there was downregulated GRP94 in lateral ventricle CP. In aged controls there was abundant staining in the epithelial cytoplasm and stroma (Fig. 2, top left). However in AD there was a striking decrease in immunostaining of GRP94 choroidal tissues (Fig. 2, top right). GRP94 is an atypical HSP in responding specifically to glucose deprivation rather than to generalized intracellular oxidative stress. In AD the opposite responses in GRP94 expression by CP vs. brain are interesting but not unexpected because secretory epithelium has biochemical characteristics fundamentally different from neurons. GRP94 chaperones protein folding, especially in endoplasmic reticulum [43]. Underexpressed GRP94 in CP of AD subjects may render the reticulum vulnerable to unfolded proteins.

In the case of HSP90, the reverse pattern of GRP94 was observed. There was faint staining of non-AD tissues (Fig. 2, bottom left) but strong expression in epithelial cytoplasm in AD CP (Fig. 2, bottom right). Multiple effects can be induced by a particular HSP. By complexing with several intracellular protein kinases, HSP90 could alter CSF secretion; and by inducing the heme-regulated e1F-2 alpha kinase, the overexpressed HSP90 may downregulate gene transcription [44]. Another possible effect of HSP90 is to beneficially accelerate clearance (reabsorption) of Aβ peptide by CP. Such facilitation occurs in the microglial handling of Aβ [45]. It would be worthwhile to pursue the role of CP HSPs in removing Aβ from CSF.

#### FGF peptides and receptors

The FGF superfamily of peptides and its multiple receptors in CP, ependyma, and brain, modulate many actions on neurons and non-neural cells [30]. FGF2, or basic FGF, is prototypic of the family. CP synthesizes and releases FGF2 into CSF. Choroidally-secreted FGF2 stimulates receptors (FGFr) nearby in the CP apical membrane [9] and at more distant sites in the brain parenchyma [46]. FGF/FGFr is apparently unique among growth factors in directly effecting balance in the brain fluids, including the formation of CSF. This is relevant to AD in which brain FGF is increased [47] and CSF turnover declines [1,4,6].

FGF and FGFr are also fundamentally important in fostering neuron generation from stem cells in the subventricular zone (SVZ). This requires coordination between the CP-CSF and periventricular regions [46,48]. Pharmacological manipulation of SVZ stem cells is potentially important at all stages of life. For pathological and therapeutic reasons, therefore, it is important to delineate FGFr expression patterns and their significance in *i) *ontogeny, *ii) *normal adult maintenance, and *iii) *neurodegeneration.

##### i) Ontogeny

Receptor plasticity in aging and AD is better seen in light of information on FGF/FGFr expression dynamics in early life. In the fetus the formation of CP, neuronal stem cells and brain is promoted by CP growth factor secretion and CSF distribution [46,49]. Intense activity of FGF/FGFr figures prominently in CNS viability and expansion. Expression of FGFr-2 and -3 in murine CP is maintained prenatally, whereas FGFr-1 and -4 are present during the 2^nd ^but not 3^rd ^gestational week [49]. FGF peptides released from CP use autocrine and paracrine mechanisms to stimulate various forms of FGFr expressed by CP epithelium. Specific functions of the four different receptor isoforms need elucidation. Despite limited data for CP FGFr expression patterns in aging, the genetic regulation of FGFr during embryonic life [49] suggests the potential to pharmacologically enhance FGFr expression in AD. The goal in filling these knowledge gaps about FGFr is to attain more efficacious treatment of injuries to the brain interior.

FGF2 derived from CP is also conveyed by CSF bulk flow to the fetal germinal matrix where it acts on stem cell FGFr to promote neuronal maturation [46]. By this endocrine-like mechanism, the CSF-mediated distribution of FGF2 and other peptides plays a prominent role in 'spawning' new neurons in the periventricular regions. Distorted CSF volume and flow in hydrocephalus interferes with the CSF provision of FGF2 to FGFr on stem cells in the SVZ [46]. Brain malformation ensues. Clearly the orderly function of the CP-CSF system, e.g., the programmed secretion and distribution of growth factors, is essential to normal CNS development.

##### ii) Adult maintenance and response to stressors

The FGF/FGFr system also has a key role in adult CNS fluid homeostasis. CP helps the brain adapt to alterations in blood composition and flow. In otherwise healthy young adults, the imposition of dehydration or sudden ischemia upon the CNS elicits striking adaptive changes in CP epithelium. To endure insults by chemical or physical stressors [29], it is critical that CP viability be maintained so that the brain can continue to benefit from 'homeostatic adjustments' in transport phenomena at the blood-CSF barrier [50]. Growth factors are an integral part of these adaptive responses to stress. Dehydration and ischemia are common to aging and AD. Elucidation of CP-CSF growth factor responses to these disorders in normal adults should enhance our perspective on homeostatic capabilities in AD.

**Figure 3:**
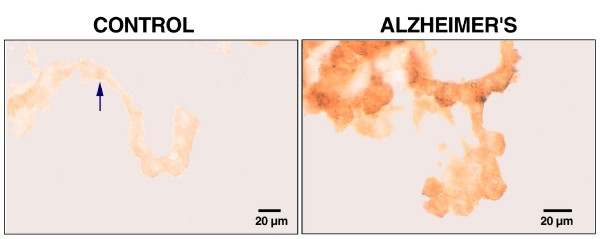
FGF receptor expression in human CP: Lateral ventricle plexuses obtained from pathologically-confirmed AD subjects were immersion-fixed in paraformaldehyde, cryoprotected and stored at -70°C. CP segments were free-floated for 72–96 hr in a 1/500 polyclonal antibody against the FGFr, which recognizes all FGF receptor subtypes. Specific staining was established by antibody omission/preabsorption [36]. ABC peroxidase/diaminobenzidine technique was used. Arrowhead points to small punctated dots of immunoreactivity. Localization of FGFr is described in text. Images are typical of those obtained from 8 controls and 8 AD specimens, respectively.

Dehydration seriously threatens CNS functions. Adjustments to dehydration in the healthy adult brain feature ion and water redistribution among fluid compartments. These compensatory responses to plasma hyperosmolality stabilize neuronal and interstitial volumes. Brain 'barriers' or transport interfaces are sites for the fluid homeostatic mechanisms. A working model for the restoration of fluid balance is offered: Dehydration or hyperosmolality upregulates the FGF2 and AVP peptides in CP [51,52]. FGF2 released by CP binds in an autocrine manner to FGFr. Such FGFr stimulation likely promotes AVP release from CP epithelium [9]. The extruded AVP then binds V1 receptors in CP to regulate ion transport [53] and fluid production [10,54]. FGF2 works in concert with AVP to control fluid movement across the blood-CSF barrier [51,55]. Interestingly in AD there are upregulated receptors for FGF (Fig. 3) and AVP [56] in human CP, presumably in response to fluid imbalance. Cumulative evidence points to co-localized growth factors and neuropeptides jointly stabilizing brain fluids after perturbed osmolality and volume.

Ischemia is another disorder with neuropathological consequences that are mitigated by growth factor upregulation or administration [57]. Bilateral carotid artery occlusion in young adult rats for 6–10 min wreaks damage to tissues surrounding the lateral ventricles [58,59]. Hence severe transient forebrain ischemia (TFI) injures the lateral plexus as well as the hippocampus [60]. However peptides such as FGF2 and TGFβ defend the forebrain interior against ischemic and hypoxic insults [57,58,61]. Although TFI with hypotension (40 mmHg) destroys many choroidal epithelial cells [60], there is a role by growth factors to efficiently repair the breached blood-CSF barrier [60]. Restitution of the epithelial lining of the choroidal villi within several hours post-stroke [60] implies the importance of a functional CP for CNS viability. Upregulated secretion of FGF2, TGFβ [58] and other growth factors by CP [27] undoubtedly protects the blood-CSF barrier and periventricular brain against compromised blood flow. A time-course analysis of FGF2-FGFr expression in the aging vs. diseased CP will reveal how the blood-CSF interface responds to reduced blood flow in NPH and AD [62,63].

##### iii) Neurodegeneration

FGF2 titers and FGFr receptor densities in degenerating CNS compartments provide insight on adaptive responses. FGF2 concentration is augmented in the AD brain [47]. Moreover with immunostaining and ELISA it was demonstrated that FGF2 levels in CP are sustained in AD [36]. It is thus probable that FGF2 and other factors secreted into CSF of aged adults are essential to forestalling harm to neurons in ischemia. A CSF feedback control system for the choroidal production of FGF2 has been suggested by FGFr identification in young adult rat CP [9]. An elevated level of FGF2 in AD brains [47] is interpreted as peptide sequestration from CSF [36], thereby lowering CSF concentration. Diminished CSF FGF2 could cause a compensatory increase in CP FGFr expression. Testing this postulate, we found enhanced staining for FGFr in AD CP epithelium (Fig. 3). This observation supports a role of the CP-CSF system in responding to increased demands by brain for FGF2. To build this model, information is needed for FGF2 and FGFr isoforms in various regions of the CNS and CSF at specific stages of dementia.

FGF2 has an interesting relationship with amyloid. A worthwhile goal is to probe mechanisms of FGF2 interaction with amyloid in neuronal networks, extracellular matrix and CP-CSF. FGF2 co-localizes with several chemical forms of amyloid. Neuronal coexistence of FGF2 and amyloid precursor protein (APP) [64,65] intimates a functional relationship between FGF2 and APP, perhaps in post-injury regeneration. FGF2 also minimizes metabolic injury caused by Aβ peptides. It was initially observed that FGF2 applied to cultured neurons reduced neurotoxicity of aggregated Aβ [66]. More recent findings confirm FGF2's benefit in abolishing neurotoxicity produced by Aβ_1-43 _[67] and attenuation of oxidative stress in hippocampal neurons induced by Aβ peptides [68]. In the extracellular matrix FGF2 competes with Aβ and APP for binding sites on heparan sulfate proteoglycans [69,70]. This competitive binding by FGF2 may suppress interstitial amyloid plaque formation. Because the interstitium receives FGF2 from CSF, we predict that pharmacological boosting of CP secretion of FGF2 would relieve AD.

FGF2-mediated protective regulation of CP transport phenomena potentially affects the course of neurodegeneration. Considerable evidence points to CP's ability to remove Aβ from CSF [71-73]. This implicates reabsorptive transport at the blood-CSF barrier to reduce CSF Aβ burden in advanced AD. In clearing Aβ from the CNS, the CP epithelium is exposed to substantial amounts of Aβ with the potential to curtail energized ion transport and fluid formation. CP Na-K-ATPase, a key enzyme in CSF production [74], also enables the transport of organic compounds [13]. Significantly, FGF2 lessens the toxicity of Aβ on Na-K-ATPase activity and mitochondrial function in cultured hippocampal neurons [68]. Toxic Aβ loads on CP transporters might impair secretion. The resultant decrease in CSF volume transmission and turnover would further destabilize the CNS. However treatment of AD with FGF analogs and IGF-1 [75] holds promise for countering Aβ toxicity [68] by creating a better CSF 'metabolic environment' for CP and brain.

#### Fluid-regulating proteins

The diminished ability of CP to form fluid in advanced ageing and AD begs the question of how epithelial ion transport proteins are altered by distorted neurochemistry in senescence. In very old laboratory mammals the Na-K-ATPase activity of CP and the CSF generated by it are cut in half [26,76]. Another ion-translocating protein coupled to CSF formation is the apical NaK2Cl cotransporter isoform1 (NKCC1). NKCC1 transports Na, K and Cl into and out of the choroidal epithelium [77], depending upon ion gradients and hormonal modulation. Versatile bidirectional transport via NKCC1 confers flexibility for regulating ion movements and concentrations in the CP-CSF. Fluid secretion at the blood-CSF interface is linked to ion fluxes mediated by the loop-diuretic sensitive NKCC1 [78,79]. NKCC1 information is plentiful for laboratory animal CP-CSF [78-84] but scarce for the human counterpart.

The cation-Cl superfamily of cotransporters includes NKCC1 and consists of 7 isoforms that actively transport Na and/or K electroneutrally with Cl [85]. NKCC1 in CP has several functions, i.e., to regulate epithelial [Cl], stabilize CSF [K] and control fluid secretion [77]. The T4 antibody differentially stains the NKCC1 secretory isoform in the apical membrane but not the KCl isoform at the basolateral surface of CP. In brain fluid homeostasis the expression of NKCC1 in CP is likely sensitive to perturbations in CSF osmolality, choroidal epithelial cell volume/ ion concentrations, intracranial pressure and ventricular volume. To shed light on compensatory responses by the CP to disease, our group analyzed NKCC1 expression in congenital, high-pressure hydrocephalus; and in adult chronic, closer-to-normal pressure hydrocephalus in AD/NPH syndromes.

**Figure 4:**
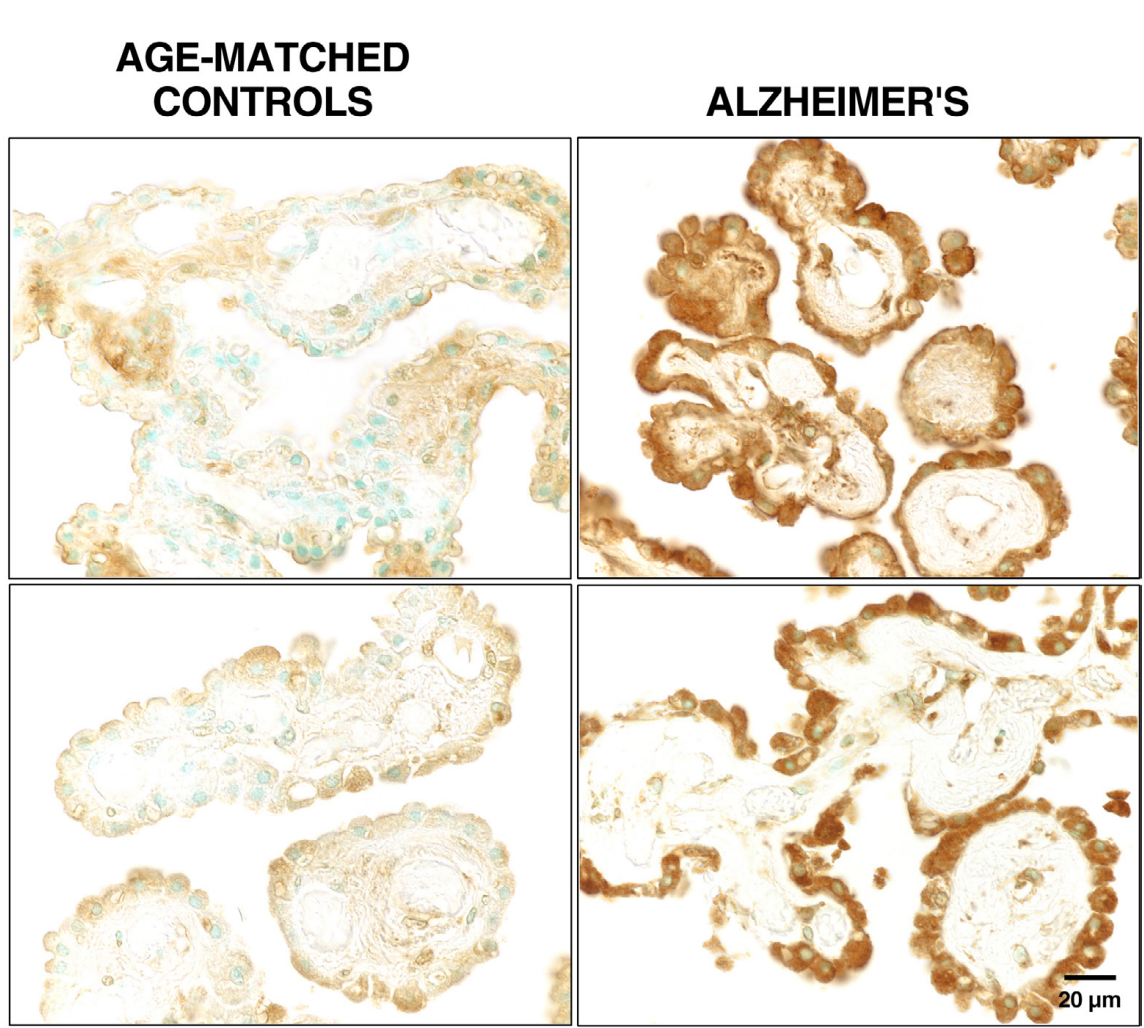
NaK2Cl cotransporter expression in human CP: Lateral ventricle plexuses were incubated with T4 (not thyroxine) antibody, which stains the secretory isoform 1 of the NaK2Cl (NKCC1) cotransporter protein. The T4 antibody (mouse monoclonal; 1:100) was from the University of Iowa Developmental Studies Hybridoma Bank (Iowa City, IA); the biotinylated secondary was a rat-absorbed horse antibody. Diaminobenzidine was used to develop the brown reaction product. Controls (negative staining results; not shown) involved omission of secondary and/or primary antibody. AD tissues were from patients at Braak stage V/VI (top right) and III/IV (bottom right). Images are representative of 6 CPs analyzed for AD (mean age of 76 yr) and 6 for age-matched controls (mean age of 76 yr). On average, the staining intensity of AD specimens was 50% greater than controls. The text describes staining localization. All photographs are at the same magnification.

The NKCC1 helps cells and organs adjust to disrupted fluid balance. One thus expects homeostatic upregulation of this CP cotransporter in AD with its altered CSF dynamics. For delineating NKCC1 expression we used the T4 antibody to immunostain CP at various stages of AD dementia. Robust staining of the lateral ventricle plexus (even at Braak stage V/VI) occurred in the apical membrane and cytoplasm (Fig. 4). In an earlier study of CP specimens from various mammalian species (unpublished data), we observed uniform and consistent staining of the apical membrane NKCC1. T4 staining of the cytoplasm was more variable. In the human CPs analyzed, however, there was consistent cytoplasmic staining in AD and age-matched controls (Fig. 4). This may represent cytoplasmic NKCC1 protein available for insertion into the apical membrane.

**Figure 5:**
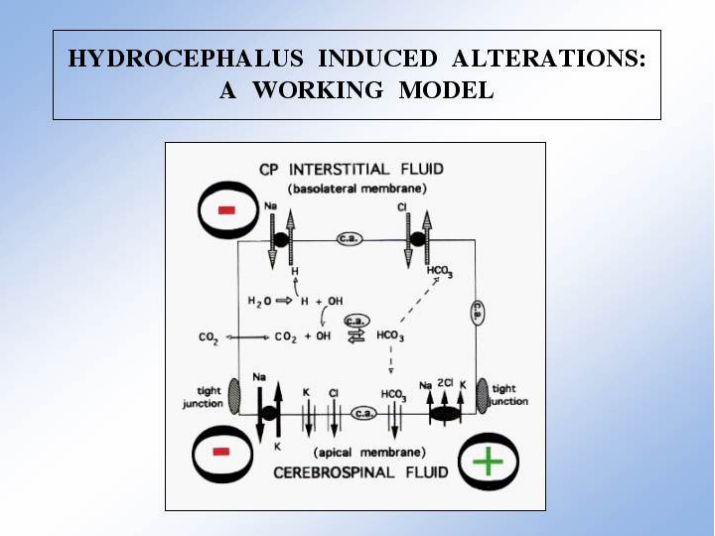
A working model for hydrocephalus-induced alterations in NKCC1 cotransporter expression in CP: Ion transport across basolateral and apical surfaces is driven by transmembrane ion gradients [74, 83]. Normally the net transport of Na, K, Cl, and HCO3 from choroid cell into CSF is integral to CSF production [5, 7]. However, we postulate that when CSF formation is inhibited by various neurohumoral agents there is stimulated (+) inward NaK2Cl flux from CSF into the cell; this would increase cytoplasmic Na and Cl concentration [77] thus creating a less favorable ion gradient for basolateral uptake of Na and Cl from plasma. Consequently, there is inhibited (-) basolateral ion uptake, and sequentially, reduced apical extrusion of Na into CSF [40]. Net effect = decreased CSF formation. Consistent with this idea are observations of enhanced expression of CP NKCCl in congenital hydrocephalus [87] and AD (Fig. 4), both of which are generally associated with lower rates of CSF formation. Agents in Table 3 (e.g., Ang II) simultaneously stimulate the inward and outward arms of NKCC1, but the former three times the latter, resulting in net inward flux of ions [discussed in ref. 93]. The model thus vectorially emphasizes the inward arm of the NKCC1 (large arrowheads) as the one primarily stimulated by agents that suppress CSF formation. We hypothesize that in hydrocephalus, with increased intracranial pressure and/or ventriculomegaly, there is an associated attenuation of fluid output by CP.

AD choroidal tissues had greater T4 staining than controls (Fig. 4). The apical NKCC1 (Fig. 5) is strategically positioned to sense physical changes in CSF resulting from AD deterioration. Changes in pressure [86] or volume represent potential stimuli for inducing NKCC1 in CP. Ventriculomegaly and transient elevations in ICP in AD and NPH may elicit a compensatory response in CP to downregulate CSF formation by promoting ion reabsorption via the NKCC1 (Fig. 5). This scheme fits the enhanced expression of NKCC1 in CP of HTx congenital hydrocephalus rats with ventriculomegaly [87]. ANP, AVP, angiotensin II, serotonin and catecholamines all reduce CSF formation rate [99]. Table 3 recapitulates the stimulating effect of these same agents on NKCC1 transport activity in various tissues [88-94]. This prompts the hypothesis that CSF formation is decreased secondarily to enhanced reabsorptive uptake of Na, K and Cl by CP *from *CSF (Fig. 5). Information about protein levels and mRNA for NKCC1 should relate CP cotransporter expression with CSF dynamics, ICP and ventricular volume.

**Table 3:** Stimulation of NaK2Cl cotransport by hormones, neurotransmitters and peptides that inhibit choroid plexus-CSF formation

*Active agent*	*Model*	*Species*	*Cotransport activity*^a^	*Reference*
Atrial natriuretic peptide	Neuroblastoma	Human	82%	88
Angiotensin II	Aortic endothelium	Cow	38%	94
Arginine vasopressin	Medullary TAL^b^	Mouse	66%	89
Serotonin agonist^c^	Fibroblasts	Cell line	49%	90
Adrenergic agonist^e^	Parotid gland epithelium	Rat	R5 staining^d^	91
Adrenergic agonist^e^	Skeletal muscle (plantaris)	Rat	1700%	92
Basic fibroblast growth factor	Aortic endothelium	Cow	40%	93

^a ^Bumetanide-sensitive ^86^Rb uptake by cells

^b ^Thick ascending limb of kidney tubule

^c ^(-)-2,5-dimethoxy-4-bromoamphetamine

^d ^R5 antibody-detected phosphorylation of NKCC1 proportional to functional activity

^e ^Isoproterenol

Other factors must be considered in interpreting the enhanced NKCC1 expression (Fig. 4). An alternative but not mutually exclusive explanation is that upregulated NKCC1 in AD helps to counter cell shrinkage [95] in CP (Table 2). Moreover, a rise in CSF [K] resulting from neuronal damage, would be buffered by the NKCC1 [77] and Na pump [96] in CP. Therefore it is possible that NKCC1 in CP is concurrently carrying out several physiologic responses to stresses imposed by aging and disease. The ventriculomegaly and reduced CSF formation rate observed in AD and NPH [4] are consistent with the upregulated NKCC1 in human CP. To corroborate the model, however, more data are needed for humans and animals to tightly link alterations in CP transport and fluid turnover with AD progression. Because HSP90 binds to NKCC1 and modulates its function [97], it is also of interest to explore how upregulated HSP90 in AD (Fig. 2) mechanistically relates to enhanced expression of NKCC1 (Fig. 4).

### The choroid plexus as a 'bioreactor' to brain diseases

CP is highly equipped, homeostatically speaking, to help the brain adapt to the metabolic distortions of dementia. Injured neurons undergo compositional changes. These cellular perturbations are transmitted to the extracellular space and distort the interstitial fluid composition. Many catabolites in the interstitium eventually gain access to the ventricles where they contact the CPs. By accepting a host of CSF-borne molecules, for either transport or receptor stimulation, the CP mediates a wide scope of renal- and hepatic-like activities [13,98]. This epithelial interface also integrates many neurohumoral activities of the endocrine and immune systems [98,99]. Diseases afflicting the CNS generate injury metabolites and cytokines that are conveyed to the ependyma, pia-glia, arachnoid membrane and CP epithelium. These interfaces handle catabolites and peptide fragments [100] by reabsorbing them into the systemic circulation for clearance or by sequestering toxic substances in lysosomes for metabolic conversion. Harmful substances are thereby effectively removed from the brain-CSF system.

Signaling molecules are also carried by CSF from diseased regions to the CP. There they bind to specific receptors in the apical membrane and consequently elicit a variety of bioreactive responses. Binding sites for a wide array of peptides, proteins and other organic substances abound in the mammalian CP [73]. Choroidal epithelial cells can thus be regarded as 'bioreactors' that respond to chemical changes in extracellular fluid. Their response includes the synthesis of peptides, growth factors and sundry molecules for homeostatically repairing injured neurons.

Currently, little is known about the spectrum of responses by CP to the disrupted CNS homeostasis in AD. It would be informative to compare AD stages for expression abilities of CP vs. the ependyma and meninges. Differences as well as similarities are anticipated. The initial studies of gene expression in human CP reported herein reveal that in Braak stages V/VI there is still strong expression of HSP90 and NKCC1 (Figs. 2 &4). Therefore even though the CP in advanced AD shows extensive histopathology (Table 2) and has reduced enzymatic activities and fluid formation [4,6,26], it evidently retains the ability to react to biochemical perturbations by expressing housekeeping proteins. This stabilizing effect on the blood-CSF barrier epithelium enables regulatory phenomena that ultimately support AD-stressed neurons.

Insight can be gained by investigating CSF neurochemical composition as a function of AD severity. Additionally it would be instructive to analyze how cultured CP epithelium reacts to 'pathological' CSF from patients at progressive Braak stages. The Z310 cultured cell line and primary cultures of CP are useful for such analyses [101,102]. Given the significance of CP in facilitating repair of CNS structures, it should also be fruitful to analyze in vivo gene expressions that reflect pathophysiological interactions between CP-CSF and brain.

### How can CP and the regions it nourishes be protected from AD?

Therapeutic strategies to halt CNS deterioration should include ways to defend the CP epithelium against the oxidative ravages of ageing and AD. Prolonging CP viability and work efficiency may be important in maintaining the well being of geriatric patients. Even without a cure for AD, if deleterious changes in the brain interstitium were minimized in the elderly by stabilizing the CP-CSF (as well as the BBB), then it might be feasible to prevent the early manifestations of AD (Braak stages I/II) from intensifying into the debilitating pathology of V/VI.

One class of agents with considerable potential for AD therapeutics is the growth factor group. A useful paradigm for CSF growth factors and neuroprotection has evolved from experiments on transient forebrain ischemia in rats [59,60]. The TFI experiments characterized the destructive effects of acute ischemia on the lateral ventricle CP and the nearby CA1 region of hippocampus [50]; and the time course of cell recovery (or death) in these adjacent regions protected (or not) by supplemental infusion of FGF2 via CSF prior to the ischemic insult [61]. Exogenous FGF2 administered before or after the induced ischemia lessened cell death in CA1, probably in part by stabilizing CP functions [28,50,61]. Moreover the CP substantially repaired its epithelial cell barrier by 24 hr post-TFI, even without supplemental FGF2 [60]. Collectively these findings manifest the impressive plasticity of CP to reconstitute itself after disruption; and reveal the potential therapeutic value of pharmacologically-administered growth factors to reduce harm to CP and hippocampus.

Several other growth factors synthesized by CP and secreted into CSF should be explored in translational research dealing with interacting ischemia, hydrocephalus and AD [1,4,6,28,36,50,55,103,104]. Following TFI the CP upregulates TGFβ, another peptide that helps the CNS adjust to injury [58]. Consequently choroidally-manufactured growth factors in disease states can benefit the plexus locally (by autocrine and paracrine mechanisms) and the brain more globally (by endocrine-like bulk flow of CSF). Our ischemia findings for the CP-CSF-hippocampus compartments [27,58,60] relate to AD in that reduced blood flow to the ageing CNS exacerbates AD progression. Growth factor supplements (e.g., VEGF, NGF, IGF-II & HGF) could augment viability in AD-vulnerable regions by enhancing vascularization, preventing programmed cell death or promoting stem cell conversion in the subventricular zone (SVZ). Newly-formed neurons in the SVZ might then migrate to atrophic regions to replace destroyed cells. One pharmacologic approach to stall AD onset is to administer a combination of growth factors designed to increase neuroprotection while minimizing fibrosis [55]. We theorize that an optimal regimen of growth factors and neuropeptides would restore CP function or prevent further loss of homeostatic capabilities.

### A look towards pharmacologic manipulation of CP in AD dementia

In searching for agents to modify CP epithelial protein expression, the route of delivery of the active drug is of primary importance. Unlike the brain with its impermeable microvessels, the CP readily takes up water-soluble drugs from the plasma due to the highly-permeable choroidal capillaries. Consequently water-soluble agents freely diffuse to receptors or binding sites at the basolateral surface of the epithelium. Access of blood-borne hydrophilic compounds to the CSF-side of CP however is problematic because the tight junctions and basolateral membrane impede diffusing molecules as small as mannitol [105]. Molecular sieving at the basolateral membrane restricts the permeation of hydrophilic molecules as small as urea (m.w. = 60) into the CP-CSF compartments [106].

To circumvent the blood-CSF barrier, therapeutic agents are delivered into CSF by lateral ventricle catheters in experimental animals [55] or hydrocephalic patients. Gene therapy offers the additional challenge of finding a viral vector that selectively targets the CP epithelium for transduction [107]. Timely, innovative strategies are in order to find specific ways to target and improve CP function in neurodegenerative states.

A compelling aspect of CSF translational research is to identify agents that effectively regulate fluid formation by CP. Whereas the difficulty in congenital hydrocephalus is to downregulate CSF formation, the challenge in AD is to enhance CSF turnover perhaps by accelerating fluid production as well as outflow. Augmented flow of CSF enhances 'sink action' [108,109]. This would expedite clearance of toxic molecules like Aβ out of the brain interstitial fluid into the ventricles. New agents that stimulate CP to secrete CSF more rapidly should be tested in aged animals and those with AD-phenotypes. A consistent finding of decreased CNS burdens of Aβ in animals with increased CSF turnover could spur the development of drugs for brain 'cleansing' in AD.

Another CP pathology meriting pharmacological attention is the massive fibrosis that progressively envelops the interstitium in old age. This interstitial fibrosis is even more extensive in AD [21]. Fibrosis undoubtedly impedes efficient movement of molecules between blood and CSF. It is pertinent to ascertain if therapeutic minimization of CP fibrosis in later life would permit a brisk turnover of CSF to be sustained. Attenuating the formation of Biondi bodies and other choroidal cellular inclusions (Table 2) would be a unique approach to prevent age- and disease-related curtailment of CSF production. Optimal vascular-interstitial-epithelial interactions in the CP are foundational for vigorous CSF dynamics. The longer the blood-CSF interface retains its epithelial secretory capabilities, the more successfully it can conduct homeostatic activities to ward off AD.

### Recapitulation and projections

The CP has the main responsibility for CSF homeostasis. Therefore the functional status of the blood-CSF barrier is of great consequence to the CNS. Maintaining the CSF at a stable, specialized composition is of the utmost importance to neurons. CSF is prominent in regulating brain interstitial fluid with which it exchanges nutrients and waste products. Diseases markedly affect these molecular exchanges. Maintaining healthy bidirectional transport across the CP epithelium (CSF-blood) and ependyma (CSF-brain) is thus integral to a sound brain fluid environment.

CSF macrocirculation through the ventriculo-subarachnoid system together with CSF microcirculation in the perivascular Virchow-Robin spaces [110,111] perform distributive as well as collective functions. By gathering waste products, the CSF is a quasi-lymphatic system with critical functions in excreting harmful peptides and proteins. In early life the upregulated secretions of CP play a central role in brain ontogeny by furnishing growth factors to the germinal matrix. At the end of life with disease onset or aging consequences, the CP transporters are upregulated again to rescue failing neurons by providing neurotrophic materials to CSF. Equally important, peptides in excess such as amyloid beta (Aβ) fragments in AD must be eliminated from CNS by perivascular pathways [112]. To facilitate clearance of Aβ, the continual production of CSF by CP sustains 'sink action' on the brain interstitium [109].

As the main generator of CSF, the CP has a pivotal role in helping the brain cope with the twin stressors of ageing and disease. More attention should be focused on the blood-CSF interface for pharmacologic opportunities to stave off CP dysfunction. Our findings on human CP expression of HSP90, FGFr and NKCC1 demonstrate that this epithelium in AD reacts to metabolic insults by upregulating certain proteins. This suggests that even diseased CP could respond to therapeutic agents, thus opening new vistas for treating CSF dysfunction in age-related dementias.

## Conclusions

Investigation of CP in AD is an area that is opening up. Translational research can now intensely focus on molecular factors that disable the CP to the point of reducing its ability to preserve brain integrity. Systematic CSF analyses using mass spectrometry and other cutting-edge biotechnology should generate neurochemical data specific for disease stages. New imaging approaches are essential to provide much needed functional data for CP, CSF and periventricular regions in AD patients. This should expedite the modeling of CP-CSF malfunctions and their resolution. Deeper insight into the pathophysiology of the blood-CSF transport interface will help to realize the development of novel therapeutic regimens for the AD family of diseases.

## Abbreviations

AD, Alzheimer's disease; APP, amyloid precursor protein; Aβ, beta amyloid; AVP, arginine vasopressin; BBB, blood-brain barrier; CP, choroid plexus; FGF2, basic fibroblast growth factor 2; FGFr, receptor for fibroblast growth factor; GRP94, glucose regulatory protein 94; HSP90, heat shock protein 90; NGF, nerve growth factor; NKCC1, Na-K-2Cl cotransporter secretory isoform 1; NPH, normal pressure hydrocephalus; TGFβ, transforming growth factor beta; SVZ, subventricular zone; TFI, transient forebrain ischemia; VEGF, vascular endothelial growth factor; IGF-II, insulin-like growth factor II; HGF, hepatocyte growth factor;

## Declaration of Competing Interests

The author(s) declare that they have no competing interests.

## Authors contributions

CJ had the primary responsibility of organizing and writing the review, and had NIH support (NS 27601) to do the NaK2Cl cotransporter experiments. PM carried out the NKCC1 immunostaining runs with the T4 antibody and provided interpretation of the regional stainings. RT conducted the experimental analyses of the heat shock proteins and generated figures. AS did the image processing analyses of the cotransporter expression and assisted with the literature analysis. JD contributed ideas for the altered CP-CSF dynamics in hydrocephalus and ventriculomegaly. GS has developed the model that CP-CSF malfunction exacerbates AD progression, and helped to revise the manuscript. ES was responsible for the FGFr experiments and interpreted the human CP data. All authors read and approved the final manuscript.

## References

[B1] Johanson CE, Silverberg GD, Donahue JE, Duncan JA, Stopa EG, Zheng W, Chodobski A (2005). Choroid plexus and CSF in Alzheimer's Disease: Altered expression and transport of proteins and peptides. The Blood-Cerebrospinal Fluid Barrier.

[B2] Huber JD, Egleton RD, Davis TP (2001). Molecular physiology and pathophysiology of tight junctions in the blood-brain barrier. Trends Neurosci.

[B3] Rubenstein E (1998). Relationship of senescence of cerebrospinal fluid circulatory system to dementias of the aged. Lancet.

[B4] Silverberg GD, Heit G, Huhn S, Jaffe RA, Chang SD, Bronte-Stewart H, Rubenstein E, Possin K, Saul TA (2001). The cerebrospinal fluid production rate is reduced in dementia of the Alzheimer's type. Neurology.

[B5] Johanson C, Conn PM (2003). The choroid plexus-CSF nexus: gateway to the brain. Neuroscience in Medicine.

[B6] Silverberg GD, Mayo M, Saul T, Rubenstein E, McGuire D (2003). Alzheimer's disease, normal-pressure hydrocephalus, and senescent changes in CSF circulatory physiology: a hypothesis. Lancet Neurol.

[B7] Johanson CE, Adelman G (2004). Choroid plexus and volume transmission. Encyclopedia for Neuroscience.

[B8] Chodobski A, Loh YP, Corsetti S, Szmydynger-Chodobska J, Johanson CE, Lim Y-P, Monfils PR (1997). The presence of arginine vasopressin and its mRNA in rat choroid plexus epithelium. Mol Brain Res.

[B9] Szmydynger-Chodobska J, Chun ZG, Johanson CE, Chodobski A (2002). Distribution of fibroblast growth factor receptors and their co-localization with vasopressin in the choroid plexus epithelium. Neuroreport.

[B10] Chodobski A, Szmydynger-Chodobska J, Johanson CE (1998). Vasopressin mediates the inhibitory effect of central angiotensin II on cerebrospinal fluid formation. Eur J Pharmacol.

[B11] Brinton RD, Monreal AW, Fernandez JG (1994). Vasopressin-induced neurotrophism in cultured hippocampal neurons via V1 receptor activation. J Neurobiol.

[B12] Moalem S, Percy ME, Andrews DF, Kruck TP, Wong S, Dalton AJ, Mehta P, Ferdo B, Warren AC (2000). Are hereditary hemochromatosis mutations involved in Alzheimer's disease?. Am J Med Gen.

[B13] Spector R, Johanson CE (1989). The mammalian choroid plexus. Sci Amer.

[B14] Serot JM, Christmann D, Dubost T, Bene MC, Faure GC (2001). CSF folate levels are decreased in late-onset AD patients. J Neural Trans.

[B15] Redzic ZB, Segal MB, Gasic JM, Markovic ID, Isakovic A, Rakic LM (2000). The kinetics of tiazofurin uptake by the isolated perfused choroid plexus of the sheep. Methods Find Exp Clin Pharmacol.

[B16] Gibbs JE, Jayabalan P, Thomas SA (2003). Mechanisms by which 2', 3'-dideoxyinosine (ddI) crosses the guinea-pig CNS barriers; relevance to HIV therapy. J Neurochem.

[B17] Tu GF, Aldred AR, Southwell BR, Schreiber G (1992). Strong conservation of the expression of cystatin C gene in choroid plexus. Am J Physiol.

[B18] Banks WA, Kastin AJ, Huang W, Jaspan JB, Maness LM (1996). Leptin enters the brain by a saturable system independent of insulin. Peptides.

[B19] Walsh RJ, Slaby FJ, Posner BI (1987). A receptor-mediated mechanism for the transport of prolactin from blood to cerebrospinal fluid. Endocrinology.

[B20] Johanson C, Del Bigio M, Kinsman S, Miyan J, Pattisapu J, Robinson M, Jones H (2001). New models for analyzing hydrocephalus and disorders of CSF volume transmission. Br J Neurosurg.

[B21] Serot JM, Bene MC, Faure GC (2003). Choroid plexus, aging of the brain, and Alzheimer's disease. Front Biosci.

[B22] Serot JM, Bene MC, Foliguet B, Faure GC (1997). Altered choroid plexus basement membrane and epithelium in late-onset Alzheimer's disease: an ultrastructural study. Ann N Y Acad Sci.

[B23] Serot JM, Bene MC, Foliguet B, Faure GC (2000). Morphological alterations of the choroid plexus in late-onset Alzheimer's disease. Acta Neuropath (Berl).

[B24] Serot JM, Foliguet B, Bene MC, Faure GC (2001). Choroid plexus and ageing in rats: a morphometric and ultrastructural study. Eur J Neurosci.

[B25] Wen GY, Wisniewski HM, Kascsak RJ (1999). Biondi ring tangles in the choroid plexus of Alzheimer's disease and normal aging brains: a quantitative study. Brain Res.

[B26] Preston JE (2001). Ageing choroid plexus-cerebrospinal fluid system. Microsc Res Tech.

[B27] Johanson CE, Palm DE, Primiano MJ, McMillan PN, Chan P, Knuckey NW, Stopa EG (2000). Choroid plexus recovery after transient forebrain ischemia: role of growth factors and other repair mechanisms. Cell Mol Neurobiol.

[B28] Ferrand-Drake M, Wieloch T (1999). The time-course of DNA fragmentation in the choroid plexus and the CA1 region following transient global ischemia in the rat brain. Neuroscience.

[B29] Murphy VA, Johanson CE (1985). Adrenergic-induced enhancement of brain barrier system permeability to small nonelectrolytes: choroid plexus versus cerebral capillaries. J Cereb Blood Flow Metab.

[B30] Weaver CE, McMillan PN, Duncan JA, Stopa EG, Johanson CE, Hertz L (2004). Hydrocephalus disorders: Their biophysical and neuroendocrine impact on the choroid plexus epithelium. Non-Neuronal Cells of the Nervous System: Function and Dysfunction Adv Mol Cell Biol.

[B31] Speake T, Whitwell C, Kajita H, Majid A, Brown PD (2001). Mechanisms of CSF secretion by the choroid plexus. Microsc Res Tech.

[B32] Sharma HS (2000). Neurodegeneration and regeneration in the CNS. New roles of heat shock proteins, nitric oxide and carbon monoxide. Amino Acids.

[B33] Johanson CE, Boulton A, Baker G, Walz W (1988). The choroid plexus-arachnoid-cerebrospinal fluid system. Neuromethods Neuronal Microenvironment-Electrolytes and Water Spaces.

[B34] Hong-Goka BC, Chang FL (2004). Estrogen receptors alpha and beta in choroid plexus epithelial cells in Alzheimer's disease. Neurosci Lett.

[B35] Murayama S, Saito Y (2004). Neuropathological diagnostic criteria for Alzheimer's disease. Neuropathology.

[B36] Stopa EG, Berzin TM, Kim S, Song P, Kuo-LeBlanc V, Rodriguez-Wolf M, Baird A, Johanson CE (2001). Human choroid plexus growth factors: What are the implications for CSF dynamics in Alzheimer's disease?. Exp Neurol.

[B37] Anthony SG, Schipper HM, Tavares R, Hovanesian V, Cortez SC, Stopa EG, Johanson CE (2003). Stress protein expression in the Alzheimer-diseased choroid plexus. J Alz Dis.

[B38] Logan A, Frautschy SA, Gonzalez AM, Sporn MB, Baird A (1992). Enhanced expression of transforming growth factor beta 1 in the rat brain after a localized cerebral injury. Brain Res.

[B39] Walter HJ, Berry M, Hill DJ, Cwyfan-Hughes S, Holly JM, Logan A (1999). Distinct sites of insulin-like growth factor (IGF)-II expression and localization in lesioned rat brain: possible roles of IGF binding proteins (IGFBPs) in the mediation of IGF-II activity. Endocrinology.

[B40] Egleton RD, Campos CC, Huber JD, Brown RC, Davis TP (2003). Differential effects of diabetes on rat choroid plexus ion transporter expression. Diabetes.

[B41] Yenari MA (2002). Heat shock proteins and neuroprotection. Adv Exp Med Biol.

[B42] Bonini NM (2002). Chaperoning brain degeneration. Proc Natl Acad Sci U S A.

[B43] Imaizumi K, Miyoshi K, Katayama T, Yoneda T, Taniguchi M, Kudo T, Tohyama M (2001). The unfolded protein response and Alzheimer's disease. Biochim Biophys Acta.

[B44] Rose DW, Welch WJ, Kramer G, Hardesty B (1989). Possible involvement of the 90-kDa heat shock protein in the regulation of protein synthesis. J Biol Chem.

[B45] Kakimura J, Kitamura Y, Takata K, Umeki M, Suzuki S, Shibagaki K, Taniguchi T, Nomura Y, Gebicke-Haerter PJ, Smith MA, Perry G, Shimohama S (2002). Microglial activation and amyloid-beta clearance induced by exogenous heat-shock proteins. FASEB J.

[B46] Owen-Lynch PJ, Draper CE, Mashayekhi F, Bannister CM, Miyan JA (2003). Defective cell cycle control underlies abnormal cortical development in the hydrocephalic Texas rat. Brain.

[B47] Stopa EG, Gonzalez AM, Chorsky R, Corona RJ, Alvarez J, Bird ED, Baird A (1990). Basic fibroblast growth factor in Alzheimer's disease. Biochem Biophys Res Commun.

[B48] Hayamizu TF, Chan PT, Johanson CE (2001). FGF-2 immunoreactivity in adult rat ependyma and choroid plexus: responses to global forebrain ischemia and intraventricular FGF-2. Neurol Res.

[B49] Reid S, Ferretti P (2003). Differential expression of fibroblast growth factor receptors in the developing murine choroid plexus. Brain Res Dev Brain Res.

[B50] Ferrand-Drake M (2001). Cell death in the choroid plexus following transient forebrain global ischemia in the rat. Microsc Res Tech.

[B51] Johanson CE, Gonzalez AM, Stopa EG (2001). Water-imbalance-induced expression of FGF-2 in fluid-regulatory centers: choroid plexus and neurohypophysis. Eur J Pediatr Surg.

[B52] Zemo DA, McCabe JT (2001). Salt-loading increases vasopressin and vasopressin 1b receptor mRNA in the hypothalamus and choroid plexus. Neuropeptides.

[B53] Johanson CE, Preston JE, Chodobski A, Stopa EG, Szmydynger-Chodobska J, McMillan PN (1999). AVP V1 receptor-mediated decrease in Cl^- ^efflux and increase in dark cell number in choroid plexus epithelium. Am J Physiol.

[B54] Faraci FM, Mayhan WG, Heistad DD (1990). Effect of vasopressin on production of cerebrospinal fluid: possible role of vasopressin (V1)-receptors. Am J Physiol.

[B55] Johanson CE, Szmydynger-Chodobska J, Chodobski A, Baird A, McMillan P, Stopa EG (1999). Altered formation and bulk absorption of cerebrospinal fluid in FGF-2-induced hydrocephalus. Am J Physiol.

[B56] Korting C, van Zwieten EJ, Boer GJ, Ravid R, Swaab DF (1996). Increase in vasopressin binding sites in the human choroid plexus in Alzheimer's disease. Brain Res.

[B57] Johanson CE, McMillan PN, Palm DE, Stopa EG, Doberstein CE, Duncan JA, Sharma HS, Westman J (2004). Volume transmission-mediated protective impact of choroid plexus-CSF growth factors on forebrain ischemic injury. Blood-Spinal Cord and Brain Barriers in Health and Disease.

[B58] Knuckey NW, Finch P, Palm DE, Primiano MJ, Johanson CE, Flanders KC, Thompson NL (1996). Differential neuronal and astrocytic expression of transforming growth factor beta isoforms in rat hippocampus following transient forebrain ischemia. Mol Brain Res.

[B59] Knuckey NW, Palm D, Primiano M, Epstein MH, Johanson CE (1995). N-Acetyl-cysteine enhances hippocampal neuronal survival induced by transient cerebral ischemia. Stroke.

[B60] Palm D, Knuckey N, Guglielmo M, Watson P, Primiano M, Johanson C (1995). Choroid plexus electrolytes and ultrastructure following transient forebrain ischemia. Am J Physiol.

[B61] Hayamizu TF, Chan PT, Doberstein CE, Sunwoo LW, Guglielmo MA, Johanson CE (1999). The role of FGF-2 in the response to cerebral ischemia: FGF-2 expression in the hippocampus and choroid plexus, and the neuroprotective effects of intraventricular FGF-2 infusion in a rat model of transient forebrain ischemia [abstract]. Soc for Neuroscience.

[B62] Edwards RJ, Dombrowski SM, Luciano MG, Pople IK (2004). Chronic hydrocephalus in adults. Brain Pathol.

[B63] Hanyu H, Shimizu S, Tanaka Y, Takasaki M, Koizumi K, Abe K (2004). Differences in regional cerebral blood flow patterns in male versus female patients with Alzheimer disease. AJNR Am J Neuroradiol.

[B64] Hagino S, Iseki K, Mori T, Zhang Y, Sakai N, Yokoya S, Hikake T, Kikuchi S, Wanaka A (2003). Expression pattern of glypican-1 mRNA after brain injury in mice. Neurosci Lett.

[B65] Imaizumi K, Iwata H, Yoshida S, Sun G, Okumura N, Shiosaka S (1993). Coexistence of amyloid beta-protein precursor and basic fibroblast growth factor in single cells of the rat parietal cortex, hippocampus and basal magnocellular nucleus. J Chem Neuroanat.

[B66] Mattson MP, Tomaselli KJ, Rydel RE (1993). Calcium-destabilizing and neurodegenerative effects of aggregated beta-amyloid peptide are attenuated by basic FGF. Brain Res.

[B67] Hashimoto Y, Niikura T, Ito Y, Sudo H, Hata M, Arakawa E, Abe Y, Kita Y, Nishimoto I (2001). Detailed characterization of neuroprotection by a rescue factor humanin against various Alzheimer's disease-relevant insults. J Neurosci.

[B68] Mark RJ, Keller JN, Kruman I, Mattson MP (1997). Basic FGF attenuates amyloid beta-peptide-induced oxidative stress, mitochondrial dysfunction, and impairment of Na+/K+-ATPase activity in hippocampal neurons. Brain Res.

[B69] Lindahl B, Westling C, Gimenez-Gallego G, Lindahl U, Salmivirta M (1999). Common binding sites for beta-amyloid fibrils and fibroblast growth factor-2 in heparan sulfate from human cerebral cortex. J Biol Chem.

[B70] Small DH, Nurcombe V, Moir R, Michaelson S, Monard D, Beyreuther K, Masters CL (1992). Association and release of the amyloid protein precursor of Alzheimer's disease from chick brain extracellular matrix. J Neurosci.

[B71] Monro OR, Mackic JB, Yamada S, Segal MB, Ghiso J, Maurer C, Calero M, Frangione B, Zlokovic BV (2002). Substitution at codon 22 reduces clearance of Alzheimer's amyloid-beta peptide from the cerebrospinal fluid and prevents its transport from the central nervous system into blood. Neurobiol Aging.

[B72] Ghersi-Egea JF, Gorevic PD, Ghiso J, Frangione B, Patlak CS, Fenstermacher JD (1996). Fate of cerebrospinal fluid-borne amyloid beta-peptide: rapid clearance into blood and appreciable accumulation by cerebral arteries. J Neurochem.

[B73] Chodobski A, Szmydynger-Chodobska J (2001). Choroid plexus: target for polypeptides and site of their synthesis. Microsc Res Tech.

[B74] Smith QR, Johanson CE (1980). Effect of ouabain and potassium on ion concentrations in the choroidal epithelium. Am J Physiol.

[B75] Carro E, Trejo JL, Gomez-Isla T, LeRoith D, Torres-Aleman I (2002). Serum insulin-like growth factor I regulates brain amyloid-beta levels. Nat Med.

[B76] Kvitnitskaia-Ryzhova TIu, Shkapenko AL (1992). A comparative ultracytochemical and biochemical study of the ATPases of the choroid plexus in aging. Tsitologiia.

[B77] Keep RF, Xiang J, Betz AL (1994). Potassium cotransport at the rat choroid plexus. Am J Physiol.

[B78] Johanson CE, Palm DE, Dyas ML, Knuckey NW (1994). Microdialysis analysis of effects of loop diuretics and acetazolamide on chloride transport from blood to CSF. Brain Res.

[B79] Johanson CE, Sweeney SM, Parmelee JT, Epstein MH (1990). Cotransport of sodium and chloride by the adult mammalian choroid plexus. Am J Physiol.

[B80] Johanson CE, Murphy VA, Dyas M (1992). Ethacrynic acid and furosemide alter Cl, K, and Na distribution between blood, choroid plexus, CSF, and brain. Neurochem Res.

[B81] Johnson DC, Singer S, Hoop B, Kazemi H (1987). Chloride flux from blood to CSF: inhibition by furosemide and bumetanide. J Appl Physiol.

[B82] Javaheri S, Wagner KR (1993). Bumetanide decreases canine cerebrospinal fluid production. In vivo evidence for NaCl cotransport in the central nervous system. J Clin Invest.

[B83] Bairamian D, Johanson CE, Parmelee JT, Epstein MH (1991). Potassium cotransport with sodium and chloride in the choroid plexus. J Neurochem.

[B84] Johanson C, Hakvoort A, Galla H-J (2001). Fluid formation by cultured porcine choroid plexus epithelium: Inhibition by furosemide and bumetanide [abstract]. Soc for Neuroscience.

[B85] Haas M, Forbush B (2000). The Na-K-Cl cotransporter of secretory epithelia. Ann Rev Physiol.

[B86] Jiang G, Akar F, Cobbs SL, Lomashvilli K, Lakkis R, Gordon FJ, Sutliff RL, O'Neill WC (2004). Blood pressure regulates the activity and function of the Na-K-2Cl cotransporter in vascular smooth muscle. Am J Physiol Heart Circ Physiol.

[B87] Johanson CE, Jones HC, Stopa EG, Ayala C, Duncan JA, McMillan PN (2002). Enhanced expression of the Na-K-2Cl cotransporter at different regions of the blood-CSF barrier in the perinatal H-Tx rat. Eur J Pediatr Surg.

[B88] Delporte C, Winand J, Poloczek P, Christophe J (1993). Regulation of Na-K-Cl cotransport, Na,K-adenosine triphosphatase, and Na/H exchanger in human neuroblastoma NB-OK-1 cells by atrial natriuretic peptide. Endocrinology.

[B89] Wu MS, Bens M, Cluzeaud F, Vandewalle A (1994). Role of F-actin in the activation of Na(+)-K(+)-Cl-cotransport by forskolin and vasopressin in mouse kidney cultured thick ascending limb cells. J Membr Biol.

[B90] Mayer SE, Sanders-Bush EL (1994). 5-Hydroxytryptamine type 2A and 2C receptors linked to Na+/K+/Cl- cotransport. Mol Pharmacol.

[B91] Flemmer AW, Gimenez I, Dowd BF, Darman RB, Forbush B (2002). Activation of the Na-K-Cl cotransporter NKCC1 detected with a phospho-specific antibody. J Biol Chem.

[B92] Gosmanov AR, Thomason DBL (2002). Insulin and isoproterenol differentially regulate mitogen-activated protein kinase-dependent Na(+)-K(+)-2Cl(-)cotransporter activity in skeletal muscle. Diabetes.

[B93] Jiang G, Klein JD, O'Neill WC (2001). Growth factors stimulate the Na-K-2Cl cotransporter NKCC1 through a novel Cl(-)-dependent mechanism. Am J Physiol Cell Physiol.

[B94] O'Donnell ME (1991). Endothelial cell sodium-potassium-chloride cotransport. Evidence of regulation by Ca2+ and protein kinase C. J Biol Chem.

[B95] Gosmanov AR, Schneider EG, Thomason DB (2003). NKCC activity restores muscle water during hyperosmotic challenge independent of insulin, ERK, and p38 MAPK. Am J Physiol.

[B96] Johanson CE, Reed DJ, Woodbury DM (1974). Active transport of sodium and potassium by the choroid plexus of the rat. J Physiol.

[B97] Simard CF, Daigle ND, Bergeron MJ, Brunet GM, Caron L, Noel M, Montminy V, Isenring P (2004). Characterization of a novel interaction between the secretory Na+-K+-Cl-cotransporter and the chaperone hsp90. J Biol Chem.

[B98] Strazielle N, Ghersi-Egea JF (2000). Choroid plexus in the central nervous system: biology and physiopathology. J Neuropathol Exp Neurol.

[B99] Nilsson C, Lindvall-Axelsson M, Owman C (1992). Neuroendocrine regulatory mechanisms in the choroid plexus-cerebrospinal fluid system. Brain Res Brain Res Rev.

[B100] Shu C, Shen H, Teuscher NS, Lorenzi PJ, Keep RF, Smith DE (2002). Role of PEPT2 in peptide/mimetic trafficking at the blood-cerebrospinal fluid barrier: studies in rat choroid plexus epithelial cells in primary culture. J Pharmacol Exp Ther.

[B101] Zheng W, Zhao Q (2002). Establishment and characterization of an immortalized Z310 choroidal epithelial cell line from murine choroid plexus. Brain Res.

[B102] Haselbach M, Wegener J, Decker S, Engelbertz C, Galla HJ (2001). Porcine choroid plexus epithelial cells in culture: regulation of barrier properties and transport processes. Microsc Res Tech.

[B103] Smith DE, Johanson CE, Keep RF (2004). Peptide and peptide analog transport systems at the blood-CSF barrier. Adv Drug Deliv Rev.

[B104] Johanson CE, Gonzalez AM, Stopa EG (2001). Water-imbalance-induced expression of FGF-2 in fluid-regulatory centers: choroid plexus and neurohypophysis. Eur J Pediatr Surg.

[B105] Johanson CE (1980). Permeability and vascularity of the developing brain: cerebellum vs cerebral cortex. Brain Res.

[B106] Johanson CE, Woodbury DM (1978). Uptake of [14C]urea by the in vivo choroid plexus-cerebrospinal fluid-brain system: identification of sites of molecular sieving. J Physiol.

[B107] Lehtolainen P, Tyynela K, Kannasto J, Airenne KJ, Yla-Herttuala S (2002). Baculoviruses exhibit restricted cell type specificity in rat brain: a comparison of baculovirus- and adenovirus-mediated intracerebral gene transfer in vivo. Gene Ther.

[B108] Hise MA, Johanson CE (1979). The sink action of cerebrospinal fluid in uremia. Eur Neurol.

[B109] Parandoosh Z, Johanson CE (1982). Ontogeny of blood-brain barrier permeability to, and cerebrospinal fluid sink action on, [14C]urea. Am J Physiol.

[B110] Rennels ML, Blaumanis OR, Grady PA (1990). Rapid solute transport throughout the brain via paravascular fluid pathways. Adv Neurol.

[B111] Rennels ML, Gregory TF, Blaumanis OR, Fujimoto K, Grady PA (1985). Evidence for a 'paravascular' fluid circulation in the mammalian central nervous system, provided by the rapid distribution of tracer protein throughout the brain from the subarachnoid space. Brain Res.

[B112] Weller RO (1998). Pathology of cerebrospinal fluid and interstitial fluid of the CNS: significance for Alzheimer disease, prion disorders and multiple sclerosis. J Neuropathol Exp Neurol.

